# Subcapsular Liver Hematoma—A Life-Threatening Condition in Preterm Neonates—A Case Series and Systematic Review of the Literature

**DOI:** 10.3390/jcm11195684

**Published:** 2022-09-26

**Authors:** Paraskevi Liakou, Anastasia Batsiou, Aikaterini Konstantinidi, Martha Theodoraki, Paschalia Taliaka Kopanou, Evangelia-Filothei Tavoulari, Andreas G. Tsantes, Daniele Piovani, Stefanos Bonovas, Argirios E. Tsantes, Nicoletta Iacovidou, Rozeta Sokou

**Affiliations:** 1 Neonatal Intensive Care Unit, “Agios Panteleimon” General Hospital of Nikea, 18454 Piraeus, Greece; 2 Laboratory of Haematology and Blood Bank Unit, “Attiko” Hospital, School of Medicine, National and Kapodistrian University of Athens, 1 Rimini Str., 12462 Athens, Greece; 3 Department of Biomedical Sciences, Humanitas University, Via Rita Levi Montalcini 4, Pieve Emanuele, 20090 Milan, Italy; 4 IRCCS Humanitas Research Hospital, Via Manzoni 56, Rozzano, 20089 Milan, Italy; 5 Neonatal Department, Medical School, Aretaieio Hospital, National and Kapodistrian University of Athens, 11528 Athens, Greece

**Keywords:** neonates, preterm neonates, very-low-birth-weight, subcapsular liver hematoma

## Abstract

The subcapsular hematoma (SLH) of the liver is a rare finding in living infants. The clinical presentation of rupture is non-specific, with the signs of hypovolemic shock dominating. The causes are several, with prematurity, trauma and sepsis playing a leading role in the creation of an SHL. Umbilical vein catheterization and an increased bleeding tendency have also been associated with this usually fatal diagnosis. Abdominal ultrasonography, among other imaging methods, comprises the gold standard examination for early diagnosis. It should be differentiated from other possible causes of shock, such as sepsis and intraventricular hemorrhage, which have similar clinical presentation. We report a case series of three very low birth weight preterms (VLBW), with an SHL, during the first days of life, one of which survived from this usually catastrophic condition. A comprehensive review of the literature regarding this clinical entity was also conducted. A high index of suspicion is essential for early identification of such a case, with conservative or surgical treatment being the way to go.

## 1. Introduction

The prevalence of preterm birth has risen over the last decades, and the global incidence now is approximately 15 million per year. Premature infants face multiple complications, with varying consequences, such as respiratory distress syndrome (RDS), sepsis, intraventricular hemorrhage (IVH), necrotizing enterocolitis (NEC), hypothermia, hypoglycemia, hyperbilirubinemia, and feeding difficulties [1]. A subcapsular hematoma of the liver (SHL) is a rare finding in living infants. It usually occurs in preterm neonates and it may be associated with birth trauma, cardiopulmonary resuscitation, sepsis, coagulopathies, maternal diseases, and placental conditions [2]. The above mechanisms can cause a simple liver laceration or even a subcapsular hematoma that can lead to a hemoperitoneum [3]. Blood accumulates under Glisson’s capsule of the liver, and this should be distinguished from intrahepatic hematoma, which occurs within the liver parenchyma [4]. In some cases, the SHL ruptures, leading to lethal massive abdominal hemorrhage, with non-specific clinical signs, which can be attributed to other clinical conditions, such as sepsis or IVH. The abdominal ultrasonography comprises the gold standard method to diagnose SHL. Singer et al. [5] report a prevalence in stillborn infants of 2.8–15%, in autopsy specimens. The purpose of our study is to present a case series of three preterm neonates with an SHL, and to report a comprehensive review of the literature on this ominous clinical entity.

## 2. Cases Presentation

### 2.1. Case 1

A 23^+4^ weeks gestation female neonate with birth weight of 770 g and product of in vitro fertilisation (IVF), first child of a 38-year-old mother, was born via a precipitous vaginal birth. Mother had antenatal care, and hypothyroidism and gestational diabetes were reported, for which she received oral medication.

At birth, the neonate was intubated and mechanically ventilated. Further resuscitation was required, chest compressions were performed, and an epinephrine bolus was administered via endotracheal tube. The neonate was admitted to the neonatal intensive care unit (NICU) and received one dose of surfactant. On clinical examination, the neonate was pale, and extensively bruised over her body and lower extremities. Her clavicle was fractured. Peripheral circulation was poor, and hypotension was recorded, requiring volume resuscitation and inotropic support.

Umbilical artery (UAC) and venous (UVC) catheters were placed. Total parenteral nutrition, antibiotics, and vitamin K (single dose—intramuscular) were administered. Hemoglobin level was low, and the neonate was transfused with red blood cells (15 mL/kg) and continuous inotropic support, with dopamine and epinephrine was required for hemodynamic stability.

**Figure 1:**
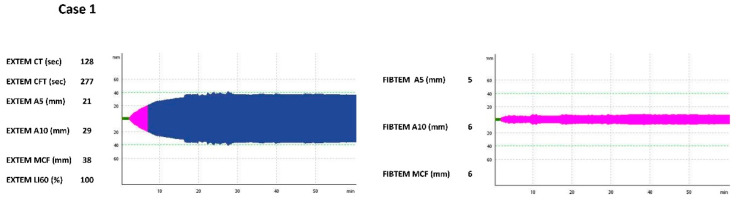
Hemostatic profile of the patient at the day of clinical deterioration, as depicted by ROTEM EXTEM and FIBTEM graphic representation and numerical results. Abbreviations: CT, clotting time (min, green colour); CFT, clot formation time (min, pink colour); A5 and A10, clot amplitude at 5 and 10 min (mm); MCF: maximum clot firmness (mm, blue colour); LI60, lysis index at 60 min (%).

On the second day of life, the clinical condition worsened, peripheral perfusion was poor, and abdominal distension and a bluish coloration of the abdominal wall were present. A heart murmur was audible. Anemia, thrombocytopenia, coagulopathy, and hypoalbuminemia were noted. Standard coagulation assays showed prolonged prothrombin time (PT), activated partial thromboplastin time (aPTT) and international normalized ratio (INR), with low fibrinogen levels. Additionally, as per our NICU protocol, rotational thromboelastometry (ROTEM—by the device ROTEM delta, Tem Innovations GmbH, Munich, Germany), was used in order to evaluate the hemostatic profile of the neonate. Extrinsically activated ROTEM (EXTEM) and fibrin-based extrinsically activated tests with tissue factor and the platelet inhibitor cytochalasin D (FIBTEM) tests were performed and a hypocoagulable state was evident by prolongation of clot formation time (CFT) in (EXTEM) assay, as well as a decrease in the clot amplitude at 5 and 10 min (A5, A10) and the maximum clot firmness (MCF) in the EXTEM and FIBTEM tests (Table 1, Figure 1). Moreover, we intended to evaluate the bleeding risk of the patient, so the neonatal bleeding risk (ΝeoBRis) score was calculated [6], which indicated a high risk of immediate bleeding (Table 1).

**Figure 2:**
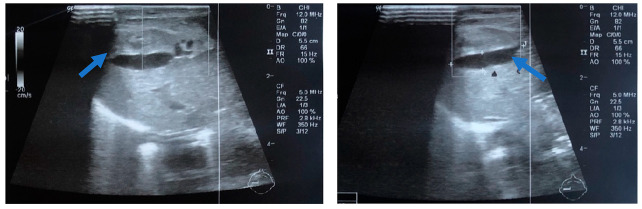
Abdominal ultrasonography depicting a cystic formation 2.1 cm × 0.5 cm in the anterior portion of the right liver, with anechoic characteristics (blue arrow).

**Figure 3:**
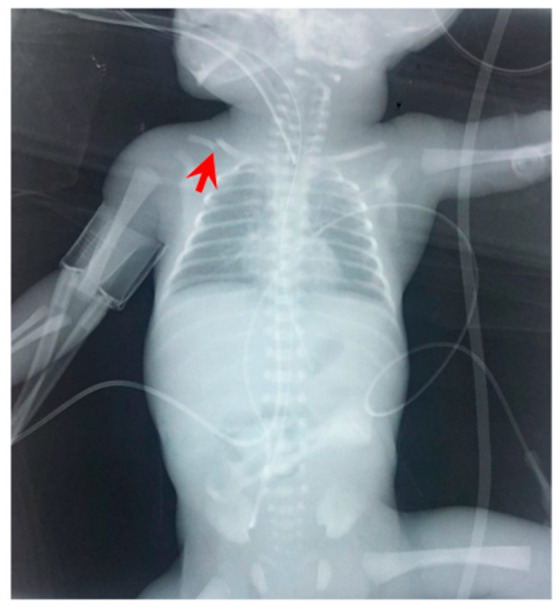
Radiography of the neonate on the day of deterioration. Non-specific signal characteristics in the abdomen, such as distention and poor distribution of bowel gas. A clavicular fracture clavicle is noted on the right (red arrowhead).

In order to differentiate the possible causes of the hypovolemic shock, the neonate had a transfontanellar brain ultrasound, which was negative for bleeding. The abdominal ultrasound revealed a cystic formation of 2.1 cm × 0.5 cm in the anterior portion of the right liver, inhomogeneous liver, and intra-abdominal fluid collection (Figure 2). Radiography of the chest and abdomen revealed abdominal distension (Figure 3). The NICU team and the pediatric surgical team, suspected a possible hemorrhage in the peritoneal cavity due to diffuse liver injury and SHL.

Based on the above clinical and laboratory findings, a crystalloid bolus, packed red blood cells and continuous inotropic support were administered. Fresh frozen plasma and vitamin K were also administered in order to control the hemorrhagic tendency. After discussion with the surgical team and based on the hemodynamic instability and poor prognosis of the patient, as the neonate was at the edge of viability, no surgical intervention was performed. Conservative treatment was continued, but the clinical status of the neonate gradually worsened. At 36 h of life, the neonate suffered pulmonary hemorrhage and on the third day of life she died.

### 2.2. Case 2

A 28^+3^ weeks gestation female infant with a birth weight of 1025 g was delivered via an emergency cesarean section, due to placental abruption, to a 35-year-old mother Gravida 2 para 2. Maternal medical history was uneventful. The delivery was complicated by difficult extraction. The neonate required resuscitation, was intubated, and transferred to the NICU. She was mechanically ventilated and received one dose of surfactant with good response. The neonate was then extubated and supported with nasal continuous positive airway pressure (nCPAP). On clinical examination the neonate had visible bruising to her body and extremities.

UAC and UVC were placed. Total parenteral nutrition and vitamin K (intramuscular) were administered, and minimal enteral feeding was started on first day of life. On the third day of life, the neonate’s clinical condition slightly worsened; laboratory findings indicative of infection were evident (elevated C-reactive protein, leucopenia), and intravascular antibiotic treatment was started (tazobactam/piperacillin and amikacin).

**Figure 4:**
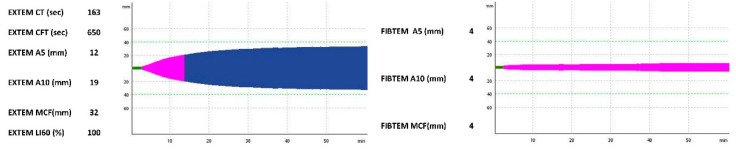
Hemostatic profile of the patient on the day of clinical deterioration, as illustrated by ROTEM EXTEM and FIBTEM graphic representation and numerical results. Abbreviations: CT, clotting time (min, green colour); CFT, clot formation time (min, pink colour); A5 and A10, clot amplitude at 5 and 10 min (mm); MCF: maximum clot firmness (mm); LI60, lysis index at 60 min (%).

On the sixth day of life, the neonate deteriorated rapidly, with paleness, poor perfusion, hemodynamic instability, abdominal distension, a palpable liver and grumble, as well as metabolic acidosis (pH: 6.96 and BE: −22.9) and acute renal failure. Hemoglobin level and platelets count were very low. Hepatic enzymes (serum glutamic-pyruvic transaminase: 2854 IU/L and serum glutamic-oxaloacetic transaminase: 291 IU/L) were elevated. A hypocoagulable profile was depicted by standard coagulation tests (prolonged aPTT and low fibrinogen levels), and ROTEM assays variables (prolonged EXTEM CFT, decreased A5, A10 and MCF in the EXTEM and FIBTEM tests; Figure 4). The 24-h bleeding risk of the neonate was calculated by NeoBris score (Table 1).

**Figure 5:**
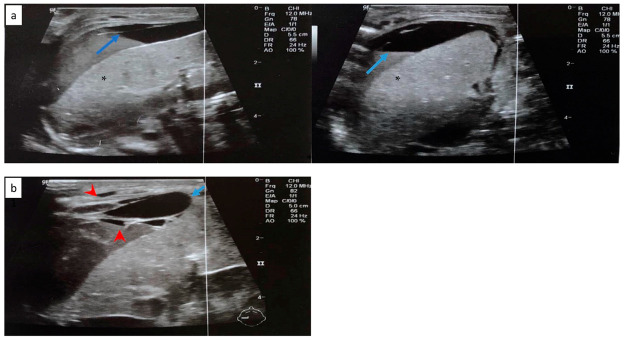
(**a**) Free perihepatic fluid collection (blue arrow) in abdominal ultrasound. (**b**) Abdominal ultrasound depicting subcapsular hematoma of the liver (blue arrow) and hemorrhagic collections in the peritoneal cavity (red arrowhead). Normal liver parenchyma is noted with asterisk (*).

**Figure 6:**
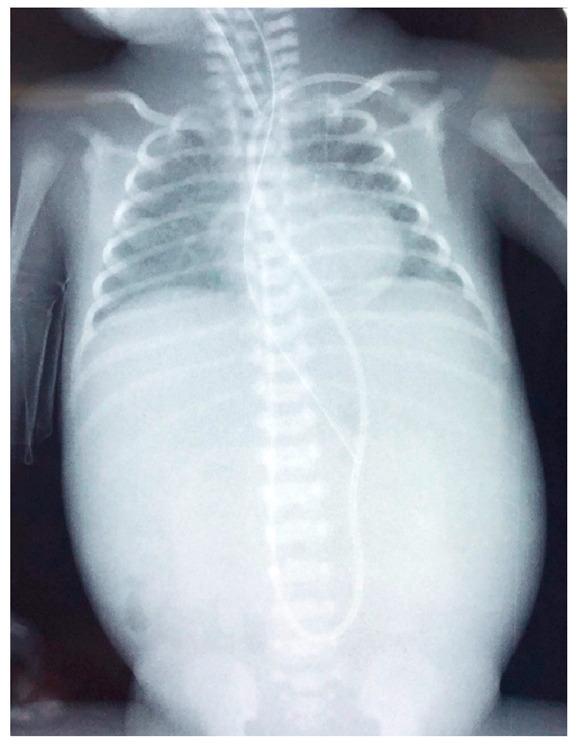
Radiography of the infant on the day of deterioration, with non-specific signal characteristics in abdomen, such as distended abdomen and poor distribution of bowel gas.

Cranial ultrasound revealed a 1st-degree IVH. Abdominal ultrasound revealed free perihepatic, perisplenic, and inter-intestinal fluid collection (Figure 5a). Abdominal distension was noted in the radiography (Figure 6).

Based on the above findings, we requested a surgical consultation. As the neonate was unstable for an exploratory research laparotomy, the pediatric surgeon team proceeded with peritoneal fluid drainage, which was hemorrhagic.

Normal saline, red blood cells and continuous inotrope agents were administered for hemodynamic support, and fresh frozen plasma and platelet transfusion for control of bleeding.

The following day, the clinical condition of the neonate deteriorated, with persistent hypotension, edema and without urine output. A second abdominal ultrasonography revealed subcapsular hematoma of the liver and the spleen and hemorrhagic collections in the peritoneal cavity (Figure 5b). The blood cultures and peritoneal fluid cultures grew *Klebsiella pneumoniae*. Despite the supportive treatment, the neonate worsened dramatically and deceased on the 8th day.

### 2.3. Case 3

**Figure 7:**
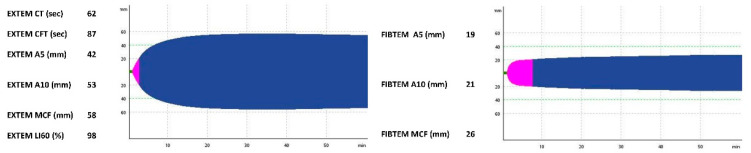
Hemostatic profile of patient on the day of clinical deterioration, as depicted by ROTEM EXTEM and FIBTEM graphic representation and numerical results. Abbreviations: CT, clotting time (min, green colour); CFT, clot formation time (min, pink colour); A5 and A10, clot amplitude at 5 and 10 min (mm); MCF: maximum clot firmness (mm, blue colour); LI60, lysis index at 60 min (%).

A 26^+6^ weeks gestation female infant with a birth weight of 900 g was delivered via a vaginal birth. The mother, a 21-year-old mother Gravida 2 para 2, did not have a maternal medical history and the pregnancy was otherwise uncomplicated. The neonate due to prematurity was transferred to NICU and was supported with nCPAP. One dose of surfactant, with less invasive surfactant administration method, was administered. UAC and UVC were placed. In the following hours the preterm was pale, and presented desaturation events, tachycardia and hypovolemia. The neonate was transfused with red packed cells, as a drop of hemoglobin was noticed. The hemostatic profile of the patient was assessed by ROTEM assays (Figure 7; Table 1). The values of ROTEM parameters fell within institutional reference ranges of thromboelastometry variables in healthy full-term and pre-term neonates [7,8], thus neither fresh frozen plasma nor PLT transfusion was administered to the patient. Additionally, the NeoBris score value of the immediate (i.e., 24 h) risk of bleeding events of the patient, was calculated as very low. We concluded that the drop of hemoglobin could be attributed to traumatic bleeding and not to congenital and/or to acquired primary coagulation disorders.

**Figure 8:**
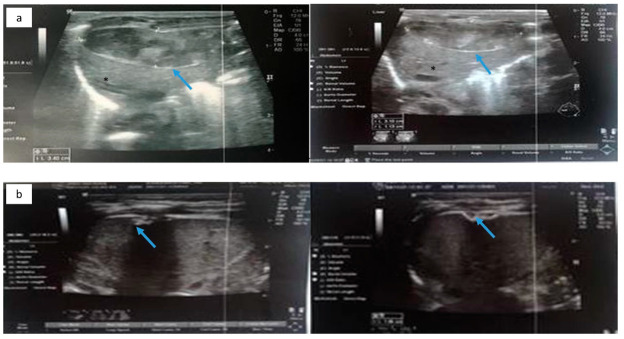
(**a**) Abdominal ultrasonography depicting hypoechogenic intrahepatic irregular lesion on the right lobe (3.4 cm × 1 cm) (blue arrow); and (**b**) abdominal ultrasonography depicting the calcific deposits (1 cm × 1 cm) (blue arrow) at the right lobe of the liver. Normal liver parenchyma is noted with asterisk (*).

**Figure 9:**
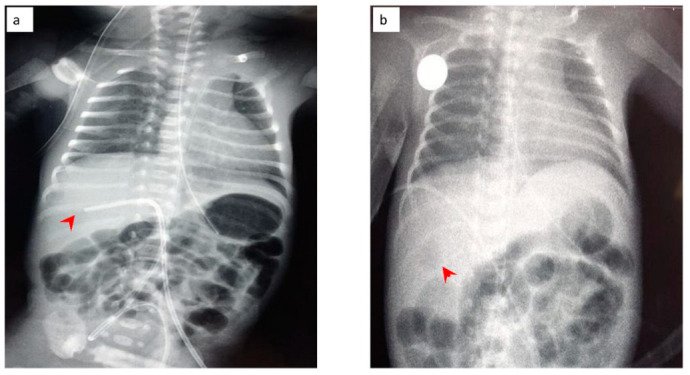
(**a**) Chest and abdominal radiography depicting the abnormal position of umbilical venous catheter in the liver (red arrowhead); and (**b**) chest and abdominal radiography depicting air in the branches of right portal vein and hepatic parenchyma (red arrowhead).

The neonate was then stabilized, and ultrasonography was performed in order to differentiate the possible causes of blood loss. No IVH was confirmed by cranial ultrasonography, while abdominal ultrasonography depicted a hypoechogenic intrahepatic irregular lesion on the right lobe (3.4 cm × 1 cm) (Figure 8a), with hematoma being the possible diagnosis from the radiologist. Chest and abdominal radiography (Figure 9a) showed an abnormal position of UVC, so it was removed. On the following radiography (Figure 9b) air in the branches of right portal vein and hepatic parenchyma was noted (as shown in Figure 9b).

The following days the neonate remained hemodynamically stable, with no evidence of new blood loss throughout the rest of her stay in the NICU. Subsequent abdominal ultrasound showed a gradual resolution of the intrahepatic lesion. Three months after the initial scan, at the right lobe of the liver, multiple hepatic calcific deposits (1 cm × 1 cm) were demonstrated (Figure 8b). The infant was discharged home on the 72nd day of life. To this date, she remains well, albeit the sequelae of his prematurity.

Family history for all the three presented cases was reported to be uncomplicated.

## 3. Review of Literature

A systematic review of the literature was conducted in September 2022. We searched the online databases PubMed and Scopus by using the combination of terms: liver subcapsular hemorrhages, subcapsular liver hematoma, subcapsular liver hematoma, neonate*, newborn*, and infant*, with Boolean logical operators (AND, OR). All studies which reported data regarding SLH cases in neonatal population were included. Studies with insufficient data, those published in congress abstract books or when only the abstract is available as well as studies published in any language other than English were excluded. Two authors (R.S., A.K.) independently screened studies for eligibility by title and abstract and then reviewed the articles retrieved in full text. In case of any disagreement, a third author reviewed the original paper in order to resolve any disagreement.

A total of 172 studies were identified from the initial search of the literature databases; 45 out of them were duplicates and were removed. After reviewing the abstract, 77 papers were excluded. Thorough reading and examination of the full text of the remaining 50 articles, led in 39 studies that met the inclusion criteria. A study flow-chart is presented in Figure S1.

A total of 39 articles reported 433 cases SLH, in neonatal population [2,3,4,5,9,10,11,12,13,14,15,16,17,18,19,20,21,22,23,24,25,26,27,28,29,30,31,32,33,34,35,36,37,38,39,40,41,42,43,44]. The characteristics of the reported cases are summarized in Table S1.

Among these 433 cases, the data regarding SLH rupture are presented for 337 cases; 199 (59.1%) out of them were associated with liver rupture and 86.6% mortality rate (Table S2);

In most of the cases (55.2%) the symptoms occurred on the first 10 days of life.

In 432 (99.8%) out of the 433 patients, antecedent events were described, such as abruptio placenta, birth asphyxia, vaginal delivery with vigorous maneuvers of neonate extraction, breech delivery, umbilical vein catheterization, indomethacin exposure for the closure of the patent ductus arteriosus and sepsis, that may have predisposed these neonates to SLH (Table S1). In a total of 433 cases, 79 (18.5%) were diagnosed intraoperatively while in 23 (5.3%) cases ultrasonography was the first choice for diagnosis and monitoring of SLH formation and resolution. In the remaining 325 (75.1%) cases the SLH diagnosis was made postmortem (Table S3).

Regarding the therapeutic choices for SLH, surgical intervention accompanied with fluid resuscitation and both crystalloid and blood products transfusion were the most frequently used (Table S3). As it concerns the coagulation status, relative data are reported only for 156 cases; thrombocytopenia presented in 127 (81.4%) cases and prolonged coagulation test in 46 (29.2%), respectively. Among the cases reported, 67 (15.5%) were stillborn, 319 (73.4%) died, while only 47 (10.9%) were survived. Preterm neonates constituted 90.4% of SLH cases, with a mortality rate of 87.9%.

## 4. Discussion

We report a case series of three incidents of SLH in preterm neonates, hospitalized in a NICU of a tertiary hospital over a 2.5-year period. The incidence of SHL was 3.6% (3 of 84) among the premature patients with very-low-birth weights (VLBW) hospitalized in our NICU over this period and account for the 20% (2 out of 10) of deaths in this population.

Hodge [44] in 1870 was the first to report a case of fatal hemorrhage from the liver in a 2 days old neonate and Rogers [20] in 1934 described the first case diagnosed during life. Since the incidence of SHL is rare, the diagnosis often is delayed or is falsely attributed to an abdominal mass or a tumor [4]. Trauma is considered to play the primary role in creating a SHL, although other causes have been described in the literature. Gennell [22] proposed that bending and compression, as in a breech presentation, of the body during birth might be the possible mechanisms of injury, causing lacerations inwards from the surface of the liver. According to the literature, the most common site of the injury is reported in the right lobe of liver, due to its anatomic position and large volume [45]. In both of the first two presented cases, a difficult birth was reported, either via a precipitous vaginal birth or a difficult extraction via a c-section. Excessive bruising on the whole surface of the neonates’ bodies was present in the first two cases. Traumatic umbilical venous catheterization and extensive infant manipulation, like cardiopulmonary resuscitation or pneumothorax decompression, are also described to cause SHL rupture [4]. The first two of our cases required resuscitation maneuvers. In the third case we hereby present, the UVC was misplaced and soon afterwards, air was noted in the branches of the right hepatic lobe, with a hypoechogenic intrahepatic irregular lesion depicted at the same area at ultrasonography. French and Waldstein reported in their study that 42% of the infants with SHL had undergone an umbilical venous catheterization [19]. According to the systematic review of the literature we conducted, preterm neonates constitute 92.4% of SHL cases. Premature and VLBW infants are more vulnerable, even without trauma; a hematoma may occur spontaneously [4]. Shankaran et al. [34] also noted that all 15 cases of SHL were in preterm VLBW infants. Our three cases refer to premature and VLBW neonates. Singer et al. [5], after conducting 755 perinatal autopsies, concluded that sepsis was associated with 62% of the cases. He proposed the initiation of the development of such lesions, by bacterial products which were responsible for creating the inflammatory process [5]. The second case suffered sepsis, with *Klebsiella pneumoniae* being isolated in the blood and peritoneal fluid cultures. Thrombocytopenia, as well as coagulopathy, such as hemophilia or Vitamin K deficiency, are common factors, although it is not clear whether thrombocytopenia is only secondary to the consumption of platelets within the hematoma [17]. Thrombocytopenia is present in 81.4% of SLH cases while prolonged coagulation test in 29.2% cases according to our systematic review. Other possible causes are poor general condition, and drugs like non-steroidal anti-inflammatory drugs [4].

The hypocoagulable profile observed in two of our cases, based on the coagulation tests we performed, enforced the fact that the hemostatic derangement is still rendered as a major contributor to morbidity and mortality of neonates with SLH and most of the cases of SLH related to coagulopathy. In our NICU, ROTEM, as a viscoelastic point of care and real time test, is regularly used in order to evaluate the hemostatic profile of neonates whenever needed, providing global information about the dynamics of clot formation, stabilization and dissolution [46]. Moreover, the NeoBris score, a ROTEM based predicting model of 24 h bleeding risk in critically ill neonates by incorporating ROTEM EXTEM variables (A10, LI60) with platelet counts and creatinine plasma levels, of our patients, showed a high risk of bleeding only in two of them. The third patient had a physiological hemostatic profile, as per her ROTEM variables, which in conjunction with NeoBris index helped us draw conclusions regarding the liver injury that occurred during UVC insertion. Additionally, in the two deceased newborns, EXTEM A10 was noted to be <37 mm and, as previously reported, [47], neonates with A10 ≤ 37 mm are 5.8 times more likely to die. Hemostasis is a dynamic process that gradually develops throughout the fetal and childhood life. Every neonate has a complex hemostatic deficiency, based on gestational age, birth weight, vitamin K reserve and grade of liver maturity [48,49,50]. Based on the existing literature, the immaturity of this mechanism is functionally compensated, and as a result, the healthy neonates, do not exhibit a hemorrhagic disposition. Although, in situations, such as infection, asphyxia or trauma, this balance is disturbed, leading to clinical conditions, such as mild or serious and even fatal hemorrhages. Hemostasis disorders are often encountered in the neonatal period, particularly among neonates admitted in NICUs and especially those with VLBW [6,51,52]. Since the premature neonate has a small total blood volume, it cannot easily compensate the hypovolemia, and falls easily into shock. Thus, prompt detection, timely intervention and appropriate management of the causes of a hemorrhage are decisive for its efficient control and patient prognosis [53]. The above-mentioned factors analyzed individually to each case, could lead to a possible time-related association between the technical manipulation and the development of the SHL.

In the cases of SHL acute rupture, the infant presents with non-specific clinical signs of abrupt massive bleeding, such as hypovolemia and shock. Neonates with slowly progressing hematomas, present with paleness, jaundice, irritability and respiratory distress [21], and since the incidence is very low, the diagnosis is usually delayed [4]. Patients are usually very premature, in a critical condition since birth [17]. When the clinical condition deteriorates, the first diagnosis that comes to mind are intraventricular cerebral hemorrhage and sepsis. The neonatologist should keep in mind this rare medical entity when the neonate presents in a hypovolemic state with abdominal distension, bluish discoloration of the umbilicus (Cullen’s sign) and scrotal ecchymosis [34]. The two first cases we reported presented with signs of hypovolemia, pallor, poor perfusion, abdominal distension, bluish coloration of the abdominal wall and a palpable liver, findings which are in line with 433 cases identified from our systematic review. Based on the differential diagnosis of the possible causes of their clinical deterioration, blood tests, intracranial and abdominal ultrasonography were performed. The patient of the third case, without rupture of liver hematoma, showed only mild signs of hypovolemia and was very easily stabilized.

Abdominal ultrasonography is the test of choice for the diagnosis of SHL, as it can picture the lesion with detail, differentiate it with other similar diagnosis, rule out rupture and help the neonatologist with the follow-up of the patient [20,21]. In all our cases, the lesions were found with ultrasonography. On abdominal ultrasonography free perihepatic, perisplenic, and inter-intestinal fluid collection can be revealed and at the same time other possible causes of hemorrhage, as IVH can be ruled out. In the case of an unruptured hematoma and the survival of the neonate, the lesions become gradually calcified as it was seen in the third case. Abdominal ultrasound also gives the neonatologist the opportunity to monitor the patient and notice the evolution of the lesion.

Management is mainly conservative including blood transfusion, correction of coagulopathies and avoidance of excessive handling of the baby, with stabilization of the infant being the main purpose [3,21]. This kind of management was followed for all our patients, although only one of them survived the hypovolemic shock. Surgical intervention may be provided for neonates who fail to respond to conservative management to improve prognosis of the ruptured SLH. Primary peritoneal drainage allows gradual release of intra-abdominal pressure, and hence the rapid enlargement of the liver, which could lead to spontaneous hemorrhage can be prevented [4,18]. Despite supportive treatment, liver rupture cannot always be avoided, having to deal with peritoneal hemorrhage. Most information regarding the techniques for management of liver hemorrhage is emerging from the trauma literature [35]. One or more of the following techniques can be used by the surgeon to control bleeding from the liver: application of topical hemostatic agents or argon beam coagulation, manual compression, suture hepatorraphy, intrahepatic vessel ligation, selective hepatic arterial ligation, partial resection, and formal hepatic lobectomy [18,36]. Our proposed algorithm for the management of a newborn with this condition is presented in Figure S2.

Although these techniques have decreased the mortality from 80% to less than 50% in these populations, they are much less helpful in neonates [21,35,36]. This is in accordance with data derived from the literature review; although 73.7% of SLH cases were treated surgically, the mortality rate remained high (89.1%). Bleeding of the liver was arrested by packing with absorbable gelatin sponge in an adult patient. Intraoperative packing with re-exploration has been advocated to manage otherwise uncontrollable liver hemorrhage in pediatric and adult trauma patients. However, the thin liver capsule can stick to the packs and worsen the injury when the packs are removed [54]. Strear et al. [35] described the novel use of thrombin and fibrin glue to control hemorrhage from the neonatal liver. The parenchyma of a neonatal liver is fragile, and the capsule is very thin, unlike that in the pediatric and adult patients [35]. Despite the considerable progress made in the surgical management of neonates the mortality still rises to 62–84.9% of infants with ruptured SHL. Surgical treatment is not usual since the patients are often unstable and should be reserved for hemorrhage of the liver and hemoperitoneum [3]. A high index of suspicion is essential to minimize morbidity as SHL is often a fatal condition.

## 5. Conclusions

SLH is a very rare condition in neonatal population and presents with no specific clinical signs but could evolve to life threatening situation. Hypovolemic shock in neonates should raise the suspicion of SLH to the clinicians, especially in case of history of perinatal trauma or manoeuvres during resuscitation. Performing an abdominal ultrasound could lead to early diagnosis of this clinical entity and guide adequate management along with monitoring of this not so usual, but fatal, situation.

## Figures and Tables

**Table 1:** The sequence of hematologic and coagulation tests performed prior and at the onset of clinical deterioration.

	Case 1		Case 2		Case 3	
Gestational age (weeks)	23^+4^		28^+3^		26^+4^	
Birth weight (g)	770		1025		900	
Gender	female		female		female	
The symptoms onset time (day of life)	2		6		1	
Clinical manifestations	Poor perfusion, anemia, hypotension, distension of the abdomen with bluish coloration		Poor perfusion, anemia, hemodynamic instability, abdominal distension		Paleness, desaturation events, tachycardia, hypovolemia	
Treatment	Conservative		Conservative, peritoneal fluid drainage		Conservative	
Outcome	Death		Death		Survived	
	**Case 1**		**Case 2**		**Case 3**	
	1st day of life	Measurements at the onset of clinical deterioration	1st day of life	Measurements at the onset of clinical deterioration	1st day of life	Measurements at the onset of clinical deterioration
WBC (K/μL)	8.67	12.74	12.89	18.41	8.94	11.91
Hb (g/dL)	11.3	5.3	14.6	4.3	14.1	6.9
PLT (K/μL)	99	48	59	17	238	153
EXTEM CT (s)	N/A	128	54	163	N/A	62
EXTEM CFT (s)	N/A	277	190	650	N/A	87
EXTEM A5 (mm)	N/A	21	26	12	N/A	42
EXTEM A10 (mm)	N/A	29	37	19	N/A	53
EXTEM MCF (mm)	N/A	38	48	32	N/A	58
EXTEM LI60 (%)	N/A	100	100	100	N/A	98
FIBTEM A5 (mm)	N/A	5	13	4	N/A	19
FIBTEM A10 (mm)	N/A	6	15	4	N/A	21
FIBTEM MCF (mm)	N/A	6	20	4	N/A	26
PT (s)	N/A	72.2	N/A	N/A	N/A	N/A
aPTT (s)	N/A	136.7	N/A	>180	N/A	N/A
INR	N/A	6.17	N/A	N/A	N/A	N/A
Fibrinogen mg/dL	N/A	43	N/A	<40	N/A	N/A
ΝeoBRis	N/A	641.3	309	704.3	N/A	81

Abbreviations: white blood count, WBC; hemoglobin, Hb; platelet, PLT; clotting time CT; clot formation time, CFT; clot amplitude at 5 and 10 min, A5 and A10; the maximum clot firmness, MCF; lysis index at 60 min, LI60; prothrombin time, PT; activated partial thromboplastin time aPTT; international normalized ratio INR; Neonatal Bleeding Risk, ΝeoBRis; not available, N/A.

## Data Availability

Data is contained within the article.

## Supplementary Materials

The following supporting information can be downloaded at: https://www.mdpi.com/article/10.3390/jcm11195684/s1, Figure S1. Study flow diagram. Figure S2. Proposed algorithm for the management of a newborn with subcapsular liver hematoma. Table S1. Characteristics of cases reported in studies included in the Review. Table S2. Review epidemiological data. Table S3. Review data regarding the diagnosis methods and treatment. References [2,3,4,5,9,10,11,12,13,14,15,16,17,18,19,20,21,22,23,24,25,26,27,28,29,30,31,32,33,34,35,36,37,38,39,40,41,42,43,44] are cited in the Supplementary Materials.
