# Supplementary material for: Subcapsular Liver Hematoma—A Life-Threatening Condition in Preterm Neonates—A Case Series and Systematic Review of the Literature
Source: J Clin Med. 2022 Sep 26;11(19):5684. doi: 10.3390/jcm11195684 (PMC9571888; doi:10.3390/jcm11195684)
Supplement: Supplementary file 1 [file jcm-11-05684-s001.zip › jcm-1915161-supplementary.pdf]

Journal of
Clinical Medicine

Supplementary Materials

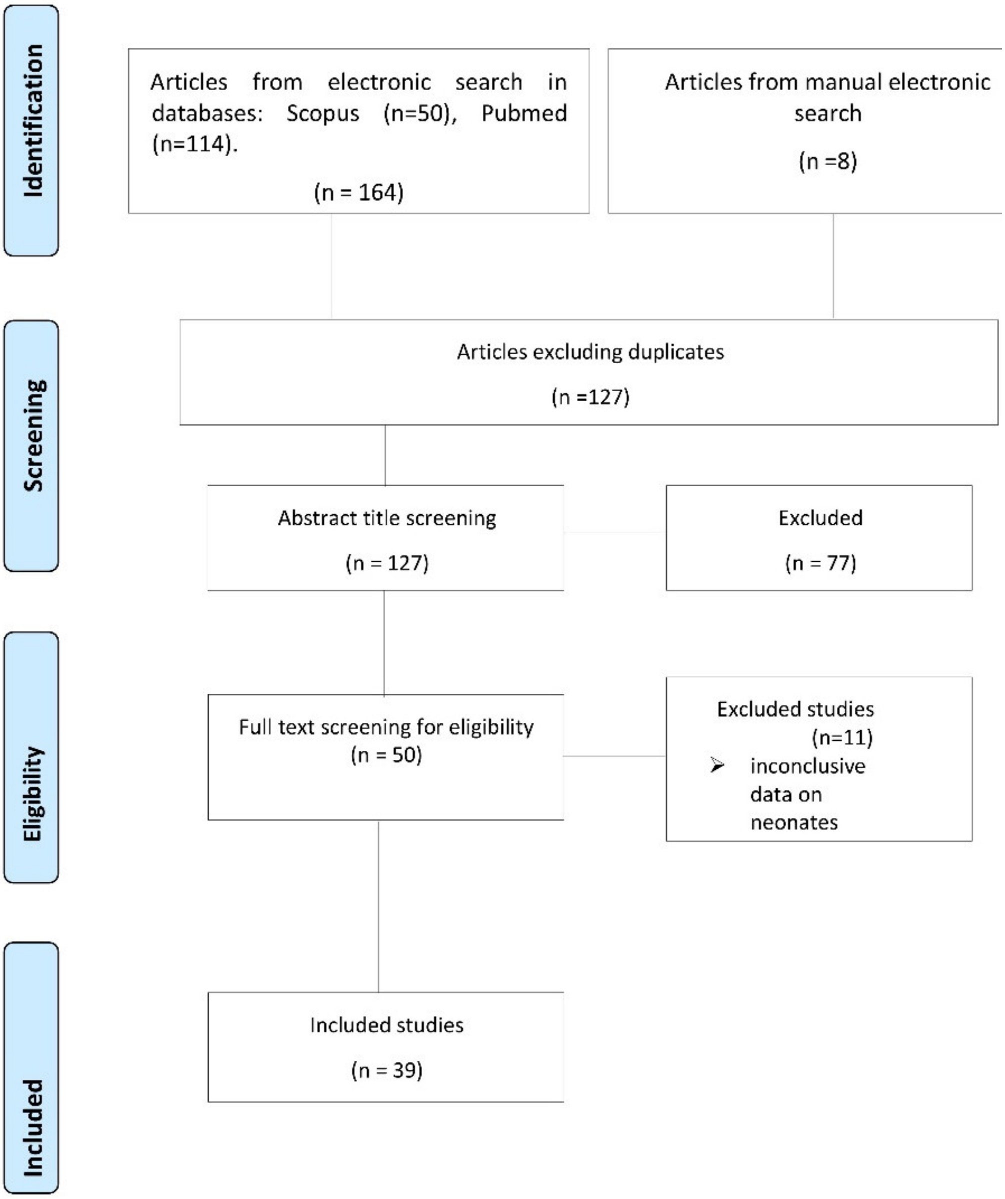

Figure S1. Study flow diagram.

Table S1. Characteristics of cases reported in studies included in the Review.

Author (Year)	Cases (N)	GA (weeks)	Symptoms Onset Time (days)	Rupture	Antecedent events	Clinical signs	Coagulation profile	Diagnostic methods	Treatment	Patient outcomes
Ahn 2010 [9]	1	29	2	No	Difficult delivery	Anemia	Platelet count decreased	Abdominal U/S	Conservative	Hematoma resolution
Amoury 1982 [10]	4	N/A	N/A	Yes	Coagulation abnormalities and evidence of sepsis.	Anemia, bradycardia, hypotension	Platelet count decreased	Abdominal U/S	Surgical	2 died and 2 survived
Anjay 2012 [3]	2	Term neonates	1-3.0	No	Birth trauma	Poor feeding, tachypnia, abdominal distension	Normal platelet count and coagulation profile	Abdominal U/S and abdominal CT	Conservative	Hematoma resolution
Buxton 1990 [11]	2	33/18	1	No	Down's syndrome and Turner's syndrome	Hydrops fetalis	N/A	Autopsy	Conservative	Death
Cewys 1967 [16]	20	N/A	1-6.0	N/A	Birth injuries	Sudden onset of pallor, followed by grunting respiration, collapsus.	N/A	Autopsy	N/A	Death
Charif 1964 [12]	41	Preterm neonates	1-8.0	Yes	Abruptio placenta, respiratory distress	Apnea, poor tone, poor feeding	Prolonged prothrombin time	Autopsy	N/A	Death
Chen 1993 [13]				in 30 cases						
Cohen 2006 [14]	1	26	11	Yes	Abruptio placenta	Anemia, abdominal distension	N/A	Abdominal U/S	Conservative	Death
Costa 2008 [15]	1	24	3	Yes	Umbilical vein catheterization	Anemia, bradycardia and hypotension	Prothrombin time slightly prolonged	Autopsy	Conservative	Death
Emma 1992 [17]	1	37	7	No	Congenital infection	Abdominal distension with pain at the palpation	N/A	Abdominal U/S and abdominal CT	Conservative	Hematoma resolution
Foss 2004 [18]	4	25-27	1-7.0	N/A	Birth asphyxia, vaginal delivery with vigorous maneuvers of neonate extraction and sepsis.	Collapsus, paleness, cyanosis and hypotension	Moderate thrombocytopenia	Abdominal U/S, autopsy	1 patient-conservative management and 3 patients-surgical intervention	Death
French 1982 [19]	1	27	7	Yes	Abruptio placenta, a transverse lie, manual version, and ultimately breech extraction, cardiac compressions	Hypotension, gastric bleeding, and acute signs of blood loss	Coagulopathy: prothrombin time1.6 sec, partial	Surgical finding	Surgical intervention	Hematoma resolution

							thromboplastin time 79 sec, fibrinogen 90 mg/dl			
Gonçalves 2013 [21]	118	22-44	<28	Yes	Respiratory distress syndrome, sepsis, congenital heart disease, pulmonary interstitial emphysema	Severe pulmonary compromise; hypoxemia, bradycardia, hypotension,	N/A	Autopsy	N/A	Death
Gruenwald 1948 [22]				in 12 cases						
Henderson 1941 [39]	1	27	3	Yes	Prematurity, very low birth weight and umbilical venous catheterization	Hypotension, tachycardia, anaemia	Thrombocytopaenia and coagulopathy	Abdominal U/S	Conservative	Hematoma resolution
Iino 1981 [23]	7	4 preterm and 3 term neonates	1-3.0	Yes	Birth injuries	N/A	N/A	Autopsy	N/A	Death
Ilhan 2017 [24]	47	N/A	1-Μαϱ	Yes in 24 cases	Asphyxia	N/A	N/A	Autopsy	N/A	Death
Kosumi 1999 [25]	1	41	1	No	Cephalopelvic disproportion	Abdominal distension	Coagulation time 8 min, bleeding time 5 min	Abdominal CT	Conservative	Hematoma resolution
Lee 2011 [26]	1	32	5	Yes	External cardiac massage	Scrotal swelling and discoloration	Normal platelet count and coagulation profile	Abdominal U/S, MRI	Conservative	Hematoma resolution
Maher 2015 [27]	1	27	2	Yes	Spontaneous vaginal delivery	Marked paleness and abdominal distention	N/A	Enhanced CT	Surgical intervention	Hematoma resolution
Maze 1979 [28]	1	38	2	No	Vaginal delivery	Scrotal swelling	Normal coagulation tests and platelet count	Enhanced CT	Conservative	Hematoma resolution
Monson 1967 [29]	1	39	Fetal life	No	N/A	No pathological findings	N/A	Fetal U/S, abdominal U/S	Conservative	Hematoma resolution
Mouratidis 1999 [30]	2	Term neonates	17 and 49	No	Large for gestational age	Petechiae and echymoses	Normal coagulation tests and platelet count	Surgical findings	Surgical intervention	Hematoma resolution
Mouzard 1982 [31]	1	Term neonate	3	Yes	Delivered by outlet forceps	Pale, cyanotic, flaccid and unresponsive	N/A	Laparotomy	Surgical intervention	Survived
Oshio 2006 [40]	1	27	3	No	Difficult umbilical venous catheter insertion	Anemia	Normal coagulation profile	Abdominal U/S and abdominal CT	Conservative	Hematoma resolution

Park 2019 [4]	16	N/A	N/A	Yes	Birth injury, prematurity with neonatal rescuscitation, particularly external cardiac massage, and epileptic mothers treated with phenobarbitone	Shock, marked anemia, dull distended abdomen	N/A	Autopsy	11 patients-conservative management and 5 patients-surgical intervention	Seven patients died without surgery; four patients survived without surgery; two patients died after surgery ; three patients survived after surgery
Rogers 1934 [20]	1	38	1	Yes	Hemophilia A	Paleness, tachypnea, abdominal distension	hepaplastin test 23.8%, antithrombin III 7.36 mg/dL, partial thromboplastin time 30 sec, and activated partial thromboplastin time>120 sec	Abdominal U/S	Surgical intervention	Survived
Ryan 1987 [32]	1	24+5	28	Yes	Premature birth with very low birth weight, breech delivery, sepsis, germinal matrix hemorrhage, indomethacin exposure for the closure of the patent ductus arteriosus	Abdominal distention, metabolic acidosis and acute renal insufficiency	N/A	Abdominal U/S	Surgical hemostasis	Death
Santos 2021 [33]	4	Term neonates	1-4.0	N/A	All neonates are characterized of excessive body size as well as difficult delivery with operative procedures	N/A	N/A	Autopsy	Conservative	Death
Shalaby 2014 [38]	6	Preterm neonates with birth weight <1000g	4.0-18	Yes	Mechanical ventilation, indomethacin treatment, birth hypoxia (in five cases), pneumothorax necessitating pleural drainage (in three cases), external cardiac massage	Marked paleness	N/A	Clinical and sonographic findings, autopsy	3 patients-conservative management and 3 patients-surgical intervention	One neonate survived

					(in three cases) and sepsis (in two cases)					
Shankaran 1991 [34]	1	Preterm neonate	1	No	Umbilical vein catheterization	Abdominal distention, scrotal discoloration, anemia, collapsus, oliguria	Thrombocytopenia	Abdominal U/S	Endovascular embolization	Hematoma resolution
Singer 1999 [5]	1	30	12	No	Appendicular abscess, NEC	Abdominal distention, anemia	N/A	Intraoperatively	Conservative	Hematoma resolution
Sokol 1974 [41]	15	27±3	1-3.0	Yes	Sepsis, resuscitation at birth	Abdominal distension, anemia	Thrombocytopenia	Autopsy	N/A	Death
Strear 1998 [35]				in 8 cases						
Theyskens 2011 [2]	52	Preterm neonates	1	N/A	Sepsis	Scrotal ecchymosis, anemia, abdominal distention	N/A	Autopsy	N/A	Death
Vanderkolk,1995 [36]	1	Term neonate	1	Yes	Severe shoulder	multiple echymoses	Grunting, pale shock	Intraoperative finding	Surgical intervention	Survived
Vachharajani 2001 [43]					dystocia, asphyxia					
Watanabe 1997 [37]	2	24-25	Окт-86	Yes	Spontaneous rupture or iatrogenic intraoperative injury	N/A	N/A	Intraoperative finding	Surgical intervention	Survived
Wolfram 1959 [42]	1	30	1	Yes	Difficult extraction of the fetus	N/A	N/A	Abdominal U/S and abdominal CT	Surgical intervention	Survived

Table S2. Review epidemiological data.

	SLH rupture	SLH	Deaths	Survivors
Cases reported	199/337 (59.1%)	433	386 (89.1%)	47 (10.9%)
Preterm	126/199 (63.3%)	206/228 (90.4%)	181/206 (87.9%)	25/206 (12.1%)
Term	10/199 (5%)	22/228 (9.6%)	9/22 (40.9%)	13/22 (59.1%)
GA not reported	63/199 (31.6%)	205/433 (47.3%)	195/428 (45.6%)	10/433 (2.3%)
Mortality	169/199 (84.9%)	386 (89.1%)		

Abbreviations: GA, gestational age; SLH, subcapsular liver hematoma. Footnotes: data are given as number of cases/total number of the data reported for each category and %.

Table S3. Review data regarding the diagnosis methods and treatment.

Diagnosis methods	n	%
Surgical finding	79	18.5
Abdominal ultrasound	23	5.3
Autopsy	325	75.1
Abdominal computed tomography	3	0.7
Fetal ultrasound	3	0.7
Total cases number	428	100
Treatment	**n**	**%**
Surgery	98	73.7
Conservative	34	25.6
Embolization	1	0.8
Total reported data	133	33.6
Not reported data	300	70
Total case number	433	100

Footnotes: the percentage (%) was calculated as the number of cases/total number of the data reported for each category and multiplied by 100.

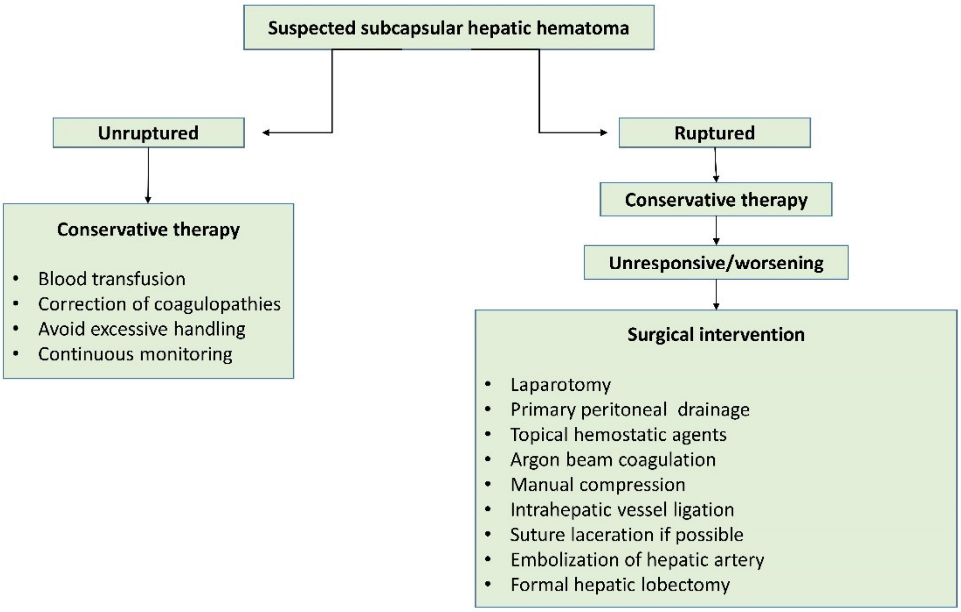

Figure S2. Proposed algorithm for the management of a newborn with subcapsular liver hematoma.
